# The deficiency of Maged1 attenuates Parkinson's disease progression in mice

**DOI:** 10.1186/s13041-023-01011-3

**Published:** 2023-02-11

**Authors:** Jie Wang, Sheng-Ye Xu, Zhi-Yuan Ye, Zhou-Na Sun, Jia-Qi Zhang, Cui Qi, Rui Liu, Xiang Gao, Chuan He, Wei-Yan You, Jun Gao

**Affiliations:** 1 Department of Neurobiology, School of Basic Medical Sciences, Nanjing Medical University, Nanjing, China; 2 Department of Rehabilitation Medicine, The Affiliated Jiangsu Shengze Hospital of Nanjing Medical University, Nanjing Medical University, Nanjing, China; 3 SKL of Pharmaceutical Biotechnology and Model Animal Research Center, Collaborative Innovation Center for Genetics and Development, Nanjing Biomedical Research Institute, Nanjing University, Nanjing, 210061 China

**Keywords:** Melanoma-associated antigen D1, Parkinson’s disease, Apoptosis, Autophagy, Substantia nigra

## Abstract

Melanoma-associated antigen D1 (Maged1) has critical functions in the central nervous system in both developmental and adult stages. Loss of Maged1 in mice has been linked to depression, cognitive disorder, and drug addiction. However, the role of Maged1 in Parkinson’s disease (PD) remains unclear. In this study, we observed that Maged1 was expressed in the dopaminergic (DA) neurons of the substantia nigra in mice and humans, which could be upregulated by the in vivo or in vitro treatment with 1-Methyl-4-phenyl-1,2,3,6-tetrahydropyridine (MPTP) or 1-Methyl-4-phenylpyridinium iodide (MPP^+^). Genetic ablation of Maged1 in mice attenuated motor deficits, the loss of DA neurons, and disease progression induced by MPTP. Moreover, Maged1 deficiency protected DA neurons against MPP^+^-induced toxicity in primary cultured cells. Mechanistically, loss of Maged1 upregulated the Akt signaling pathway and downregulated the mTOR signaling pathway in SH-SY5Y cells, which may in turn attenuate the cell apoptosis and impairment of autophagy*.* Consistent with it, the degeneration of midbrain and striatum among elderly Maged1 knockout mice was relatively mild compared to those in wild-type mice under physiological conditions. Taken together, this study suggested that Maged1 deficiency inhibited apoptosis and enhanced autophagy, which may provide a new potential target for the therapy of PD.

**Supplementary Information:**

The online version contains supplementary material available at 10.1186/s13041-023-01011-3.

## Introduction

Parkinson’s disease (PD) is the second most common neurodegenerative disease after Alzheimer disease, which affects approximately 1% of the population over the age of 60 years [1]. To date, the precise pathogenesis of PD remains unclear; the pathological hallmark of PD involves the progressive dysfunction of the nigrostriatal pathway, ultimately leading to loss of dopaminergic innervation in the striatum along with widespread intracellular α-Synuclein (α-Syn) aggregates in the substantia nigra pars compacta (SNc) [2].Although there are several drugs to treat symptoms in patients with PD, these fail to prevent disease progression. No neuroprotective therapies are currently available largely owing to the limited understanding of the pathogenesis of PD [3]. Thus, there is an urgent need to explore the precise pathogenic mechanism at the molecular level and provide effective therapy for PD.

Convincing evidence suggests that mitochondrial deficits, endoplasmic reticulum (ER) stress, and neuroinflammation play a role in the etiology of PD [4–6]. The causes of neuronal death in PD are a constant matter of controversy. Apoptosis has been considered the main mechanism of neuronal death in most neurodegenerative conditions, including PD [7]. In recent years, emerging lines of evidence suggest a close association between impaired autophagy and PD [8–11]. Autophagy plays a key role in maintaining intracellular energy and homeostasis by clearing long-lived proteins, aggregated proteins, or dysfunctional organelles. There is evidence that several PD-related genes, including *SNCA*, *LRRK2*, and *FBXO7*, are involved in the process of autophagy [12]. The balance between apoptosis and autophagy maintains intracellular homeostasis under normal conditions, while it is disturbed in PD, which accelerates neurodegeneration [13]. Therefore, restoring this imbalance between apoptosis and autophagy may be effective in treating PD.

*Maged1* belongs to the large *MAGE* family, also known as *NRAGE* or *Dlxin-1,* first described as genes expressed in cancer and germline cells [14]. The gene encodes an 86-kDa protein and is highly expressed in several tissues during developmental and adult stages, especially in the brain [15]. Maged1 mediates different functions such as differentiation, apoptosis, cell cycle, tumorigenesis, and adipogenesis, by interacting with different proteins [16]. Our previous study reveals that Maged1 is involved in synaptic transmission and hippocampus-dependent learning and memory formation by interacting with cAMP response element-binding protein [17]. As a classical molecule that regulates neuronal apoptosis through bone morphogenetic protein (BMP) signaling, p75 neurotrophin signaling, and UNC5H signaling [18–20], it appears that Maged1 may play a role in the pathogenesis of PD. Moreover, a PD-related gene FBXO7 was reported to positively regulate BMP-mediated signaling through Lys-63-specific ubiquitination of Maged1 [21].

Given the evidence cited above, we hypothesized that Maged1 might play a vital role in the pathology of dopaminergic (DA) neurons in PD. In our in vivo study, we observed that loss of Maged1 in mice attenuated motor deficits, DA neuron loss, and disease progression induced by 1-Methyl-4-phenyl-1,2,3,6-tetrahydropyridine (MPTP). In vitro, Maged1 deficiency protected the DA neurons against 1-Methyl-4-phenylpyridinium iodide (MPP^+^)-induced toxicity in primary midbrain cultures, differentiated ReNcell VM cells (a human neural progenitor cell line), and SH-SY5Y cells. Furthermore, we considered that inhibition of Maged1 may restore the imbalance between apoptosis and autophagy in PD therapy, owing to the upregulation of the Akt signaling pathway and downregulation of mTOR signaling pathway.

## Materials and methods

### Antibodies and reagents

All antibodies and reagents used in this study are listed in detail in Additional file 1: Tables S1 and S2.

### Human sample

Human brain sections were obtained from the Department of Forensic Medicine in Nanjing Medical University. The protocols for human research were approved by the Ethics committee of Nanjing Medical University (Permit Number: 2019-889).

### Animals

All animal experiments were performed according to the protocols approved by the Animal Core Facility of Nanjing Medical University (IACUC-1601153). The Maged1 KO mice used in this study were provided by the Animal Research Center (Nanjing University, China). Mice were maintained on a C57BL/6 background. Animals were housed in the Experimental Animal Center of Nanjing Medical University on a 12 h light/dark cycle. 8–10-week-old male mice were used. To establish the acute model of Parkinson’s disease, mice were intraperitoneally injected with 20 mg/kg MPTP four times, after 2-h intervals. Three days after injection, the midbrain and striatum were collected for western blot or immunohistochemistry to detect autophagy-related markers. Four days after injection, behavioral tests were performed. Seven days after injection, tissues were collected for western blot, high-performance liquid chromatography (HPLC), and immunohistochemistry.

### Genotyping

The genotype of mice was identified via PCR using genomic DNA obtained from their toes. The primers for genotyping were as follows: Maged1-WT*-*F: AGATCCTCCTCCAACTCTCG, Maged1-WT-R: GAAAAACCCCACAAGCTTACC. Maged1-KO-F: AAACCACACTGCTCGACCTAGC, Maged1-KO-R: CCAATTTAGACTCCCCCAAGACC. The program was as follows: following initial denaturation for 5 min at 94 °C, 30 cycles including denaturation for 40 s at 94 °C, annealing for 30 s at 55 °C, and elongation for 1 min at 72 °C. The products were electrophoresed on a 1% gel at 120 V.

### Behavioral tests

Behavioral tests were performed on the fourth day after MPTP injection. To examine motor balance and coordination, the rotarod test and pole test were used. For the rotarod test, after being handled for 4 consecutive days, mice were trained with 16 revolutions per minute (rpm) for 1 min on a rotarod apparatus for 3 consecutive days. Each mouse was trained three times a day. On the testing day, mice were tested on the rotarod set at 25 rpm for 60 s and their latency to fall off the rotarod was recorded. Pole test: A 55-cm gold-threaded rod with a diameter of 0.8 cm was used. Mice were placed on the top with their heads oriented upward, and the time for them to turn from the top and descend to the base of the pole was calculated to reflect their motor ability. During the training phase, mice were trained 5 times a day. On the testing day, the time for each mouse to reorient themselves facing downward of the straight bar and descend to the bottom was recorded. Each mouse was tested 5 times and the values were averaged.

### Immunohistochemistry

After the mice were deeply anesthetized using pentobarbital, cardiac perfusion was performed with 20 mL cold PBS and 40 mL pre-cooled 4% paraformaldehyde solution. The brains were transferred to 4% PFA and fixed at 4 °C for 24 h and then transferred to 30% sucrose solution (prepared with PBS) at 4 °C for dehydration until the brain tissues sank to the bottom of the centrifuge tubes. Brains were embedded in optimal cutting temperature compound (OCT) and deep-frozen at − 80 °C. Tissues were sectioned to obtain the midbrain or striatum Sections (25 µm) for immunohistochemistry. Then, the sections were immersed into the citrate antigen retrieval solution and heated for 5–10 min in a microwave. Next, the sections were blocked in PBS with 0.3% Triton X-100 and 10% FBS for 2 h at room temperature (RT). Then, the sections were incubated with the primary antibodies overnight at 4 °C and rinsed with PBS plus 0.1% Triton X-100 for 3 × 10 min the next day. The primary antibodies were washed and the sections were incubated with the corresponding fluorescent secondary antibodies and DAPI in darkness for 2 h at room temperature. After washing with PBS three times for 10 min each, and with 0.1% PBST once, sections were sealed using glycerin solution. Stained sections were observed using a laser confocal microscope (FV-1200, Olympus, Japan).

### High-performance liquid chromatography (HPLC)

Tissue levels of DA and its metabolites (DOPAC, HVA) were determined using HPLC. Briefly, the striatum of mice was homogenized in 0.1 M perchloric acid (HClO_4_) containing 100 mM disodium EDTANa_2_ and 3.5 × 10^–8^ M DHBA, and then centrifuged at 20,000 × *g* for 30 min at 4 °C. The supernatants were filtered through a 0.45-mm pore membrane and analyzed for 5-HIA, DA, and DOPAC using HPLC coupled with electrochemical detection.

### Cell culture

The SH-SY5Y cell line was a kind gift from Shi Yun (Animal Research Center, Nanjing University, China). Cells were cultured in DMEM/F12 supplemented with 10% fetal bovine serum at 37 °C in 5% CO_2_.

Primary dopaminergic neuron cultures were prepared from the ventral mesencephalon of embryonic day 13.5 (E13.5) fetal mice. Briefly, embryos were obtained and placed in Hanks’ balanced salt solution and dissected to obtain mesencephalon tissue. Next, it was treated with 0.125% trypsin for 5 min at 37 °C, followed by neurobasal medium supplemented with 10% FBS. Approximately 2 × 10^5^ cells/mL were seeded on poly-L-lysine-coated coverslips and cultured in 500 μL of neurobasal medium supplemented with 2% B27 and 0.5 mM glutamine. The medium was replaced with fresh medium after 24 h and the cells were cultured for 7 days before incubation with 200 μM MPP^+^ for 24 h. Then, the cells were characterized by immunostaining for Tuj1 and TH.

ReNcell VM cells (purchased from Millipore) were cultured as neurospheres in DMEM/F12 supplemented with 2% B27, 20 ng/mL epidermal growth factor (EGF), and 20 ng/mL basic fibroblast growth factor (bFGF). To label ReNcell with tdTomato, cells were stably transduced with packaged lentivirus vectors to express tdTomato fused with the palmitoylation sequence of growth cone-associated protein (PalmtdTomato). The plasmid was kindly provided by Dr. Bakhos Tannous (Massachusetts General Hospital, Boston, MA, USA). After 5–7 days of proliferation, aggregated cells were collected and dissociated by gentle trituration and replated on laminin-coated coverslips with media without bFGF and EGF. Differentiation was initiated by the addition of 1 mM dibutyryl-cAMP and 2 ng/mL glial cell-derived neurotrophic factor (GDNF). Experiments were conducted 12 days after differentiation.

### Plasmid and siRNA transfection

For plasmid or siRNA transfection, SH-SY5Y cells seeded in 35-mm dishes were transfected using Lipofectamine 2000 or Lipofectamine-iMAX according to the manufacturer’s instructions. Twenty-four hours post-transfection, 200 μM MPP^+^ was added for another 48 h for downstream experiments. For autophagy assessment, 24 h after transfection with siRNA-Maged1, cells were further transfected with the GFP-LC3 vector to label autophagosomes for another 24 h followed by treatment with 200 μM MPP^+^ for 48 h. Then, the cells were fixed and immunostained using anti-GFP antibody. Immunofluorescence was determined as described above. siRNA-Maged1 were purchased from GenePharma. Sequence for si-Maged1: GGUCAAGUACUUGAUGCUUTT, normal control (NC): UUCUCCGAACGUGUCACGUTT.

### Western blot

Mice were deeply anesthetized with pentobarbital and decapitated. Brains were collected and dissected rapidly on ice to obtain the midbrain and striatum, which were then frozen in liquid nitrogen and stored at − 80 °C until further use. When needed, tissues were homogenized in 300–500 μL lysis buffer containing protease and phosphatase inhibitors. The homogenates were incubated for 30 min on ice and centrifuged at 12,000 rpm for 15 min at 4 °C. The supernatant protein was collected and 5 × SDS loading buffer was added. After boiling at 95 °C for 8 min, equivalent amounts of protein (40 µg) were separated using 8–12% SDS-PAGE gels and transferred to PVDF membranes. Membranes were blocked in 5% milk in TBS with 1% Tween (TBST) for 1 h and were incubated overnight with primary antibodies diluted in TBST with 1% bovine serum albumin. The following day, after 3 × 8 min washes with TBST, membranes were incubated with secondary antibodies for 1 h at RT. After 3 × 10 min washes with TBST, protein signals were detected using an ECL chemiluminescence kit and the luminescence was visualized using a Tanon luminescent imaging system. Image J was used to obtain quantified results. SH-SY5Y cells were washed with cold PBS three times and incubated in 200 μL lysis buffer containing protease and phosphatase inhibitors for 30 min and centrifuged at 12,000 rpm for 15 min at 4 °C. Western blot was performed as described earlier [17].

### CCK8 assay

To evaluate cell viability using CCK8 assay, cells were seeded in a 96-well plate (1 × 10^4^ cells/well). After MPP^+^ treatment for 48 h, cells were exposed to fresh culture medium containing 10% CCK8 and then incubated at 37 °C for an additional 1.5 h. The absorbance was measured at 450 nm.

### Propidium iodide (PI) staining assay

Apoptosis of SH-SY5Y cells was determined using the PI staining assay and flow cytometry. Briefly, after the indicated treatment, cells were collected and stained with PI before flow cytometry analysis. About 10,000 cells were collected and analyzed using the FACS cytometer (BD Bioscience).

### Transmission electron microscopy

Collected cell pellets were fixed in sodium cacodylate buffer (0.1 M; pH 7.4), 2.5% glutaraldehyde, and 0.25 M sucrose at 4 °C, then post-fixed in 1% osmium tetroxide for 1 h at 4 °C. Samples were dehydrated, embedded in plastic, and cut into in 70-nm sections for microscopy. Sections were subsequently poststained using 5% uranyl acetate and viewed using a Tecnai G2 transmission electron microscope.

### Statistical analysis

All data are presented as mean ± SE. Statistical significance between 2 groups was calculated using a two-tailed Student’s *t-*test for parametric variables and Mann–Whitney’s *U* test for nonparametric variables. One-way ANOVA with Tukey’s posthoc tests was used for comparisons among groups. *P* < 0.05 was considered statistically significant. Randomization and blind analyses were used whenever possible.

## Results

### Maged1 is upregulated in MPTP-treated mice and MPP^+^-intoxicated SH-SY5Y cells.

**Fig. 1:**
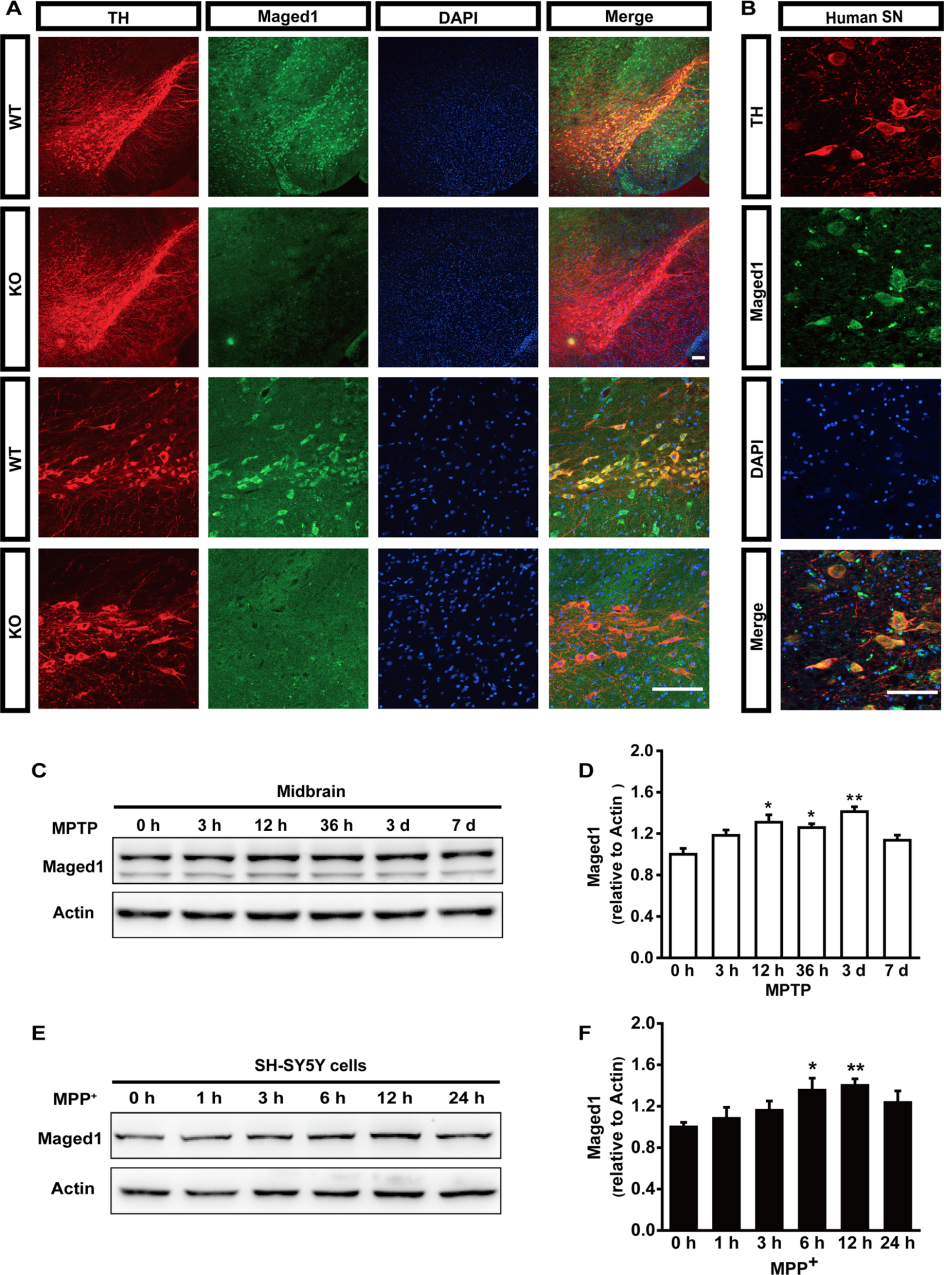
Expression of Maged1 in MPTP-treated mice and MPP^+^-intoxicated SH-SY5Y cells.** A**, **B** Confocal images showing Maged1 (green) and TH (red) colocalization in the substantia nigra of mice (**A**) or humans (**B**);nuclei were counterstained with DAPI (blue).Scale bar: 100 μm. **C**, **D** Four intraperitoneal injections of MPTP (20 mg/kg) were administered to male C57BL/6 mice at 2-h intervals daily and total protein was extracted from the midbrain of selected mice (time was calculated from the last injection of MPTP), and then analyzed using western blot to determine the protein level of Maged1.n = 3 mice per group; actin was used as a loading control. **E**, **F** SH-SY5Y cells were treated with 200 μM MPP^+^ for the indicated time, cells were harvested, and protein levels of Maged1 determined using western blot analysis. Experiments were performed in triplicate. Quantitative data are shown as means ± SE. * *P* < 0.05, ** *P* < 0.01

To determine whether Maged1 played a physiological role in PD in vivo, we first confirmed the expression of Maged1 in dopaminergic neurons by co-immunostaining Maged1 with tyrosine hydroxylase (TH), a marker of the DA neurons. Notable Maged1 immunoreactivity was observed in TH-positive neurons, which was abolished in Maged1 knockout (KO) mice (Fig. 1A). Concordantly, Maged1 also existed in the DA neurons in the SN from the human sample (Fig. 1B). We investigated the expression of Maged1 in the midbrain of MPTP-treated mice using western blot and found that Maged1 protein level was significantly increased after an MPTP challenge for 12 h, which was sustained for 3 days, then back to the normal on the 7th day (Fig. 1C, D). Similarly, a time-dependent increase of Maged1 protein level was detected in MPP^+^-treated human SH-SY5Y cells (Fig. 1E, F). Together, these results indicated that Maged1 was expressed in DA neurons, and was regulated by the neuronal toxicity, suggesting that Maged1 might have been involved in the pathogenesis of PD.

### Maged1 knockout in mice attenuates MPTP-induced motor deficits and disease progression

**Fig. 2:**
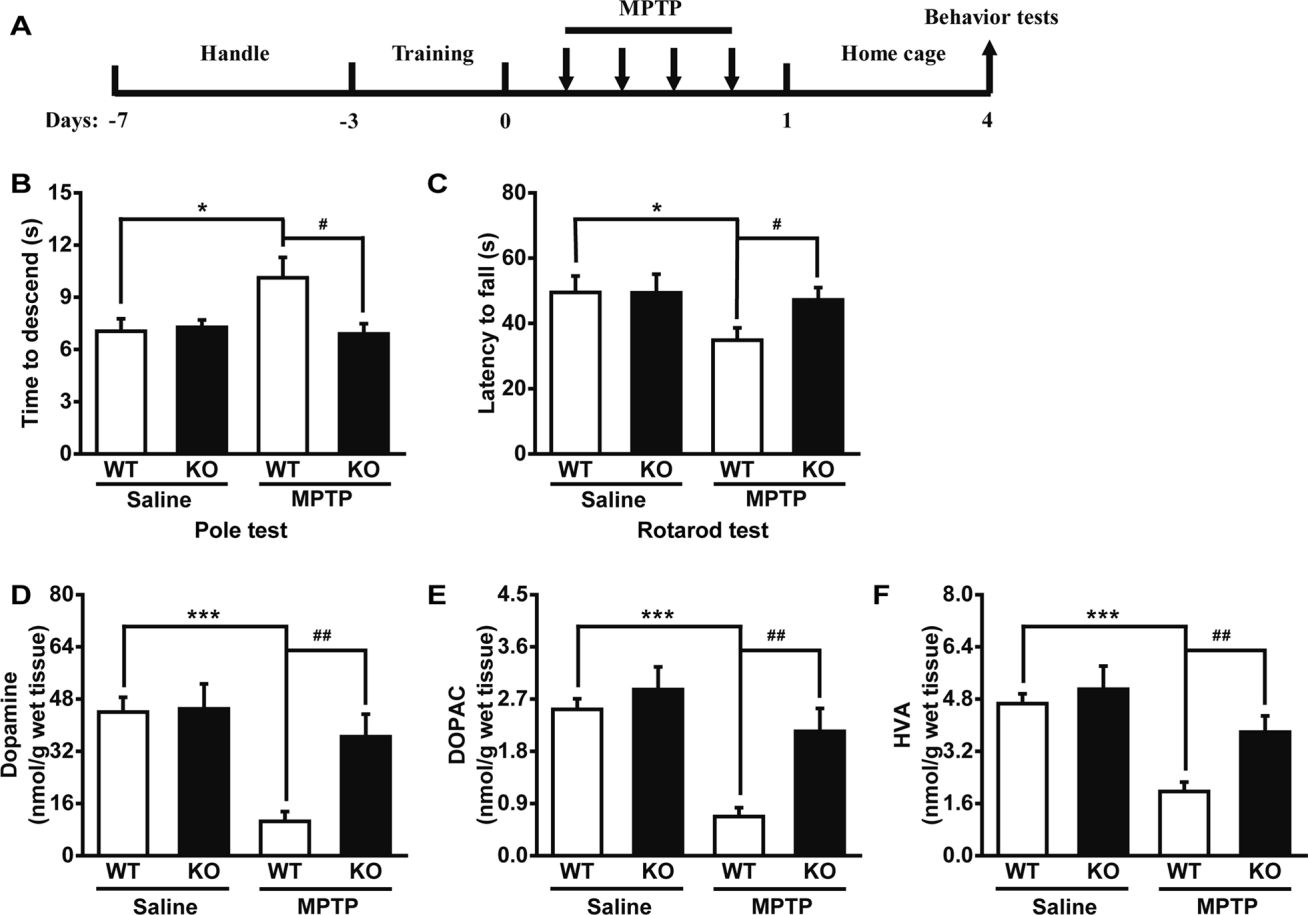
Genetic ablation of Maged1 attenuates motor deficits and disease progression induced by MPTP.** A** Schematic diagram of the experimental design for behavioral tests. **B**, **C** Quantitative analyses of motor coordination using the pole test **B** and rotarod **C** tasks, n = 7 per group. **D**, **F** Quantification of dopamine (**D**), DOPAC (**E**), and HVA (**F**) using HPLC in the striatum, n = 7 per group. Data are shown as means ± SE. *P < 0.05, ***P < 0.001, ^#^*P* < 0.05, ^##^*P* < 0.01

To determine the involvement of Maged1 in PD, we used the Maged1 knockout mouse model. Firstly, we investigated the effects of Maged1 deficit on behavior of MPTP-treated mice using the pole test and rotarod test (Fig. 2A). As shown in Fig. 2B, C saline-treated WT and Maged1 knockout (KO) mice showed no significant differences in performance on any motor coordination tasks. After MPTP treatment, WT mice took longer to pass the pole test and fell from the rotarod faster, while KO mice behaved much better than that WT mice did. Moreover, to assess the successful establishment of the neurodegeneration model, we detected the levels of striatal dopamine and its metabolites on the seventh day after MPTP administration, using high performance liquid chromatography (HPLC). Consistent with the behavioral test results, the levels of dopamine, 3,4-dihydroxyphenylacetic acid (DOPAC), and homovanillic acid (HVA) were significantly reduced in the striatum of the WT mice after MPTP injection, but which was blocked in Maged1-deficient mice. Furthermore, Maged1 knockout alone did not alter the levels of these markers (Fig. 2D–F). All these data suggested that Maged1 deficit improved the effect of MPTP treatment on the behavior and biochemical indexes in mice.

### Maged1 knockout in mice rescues DA neurons from MPTP toxicity

**Fig. 3:**
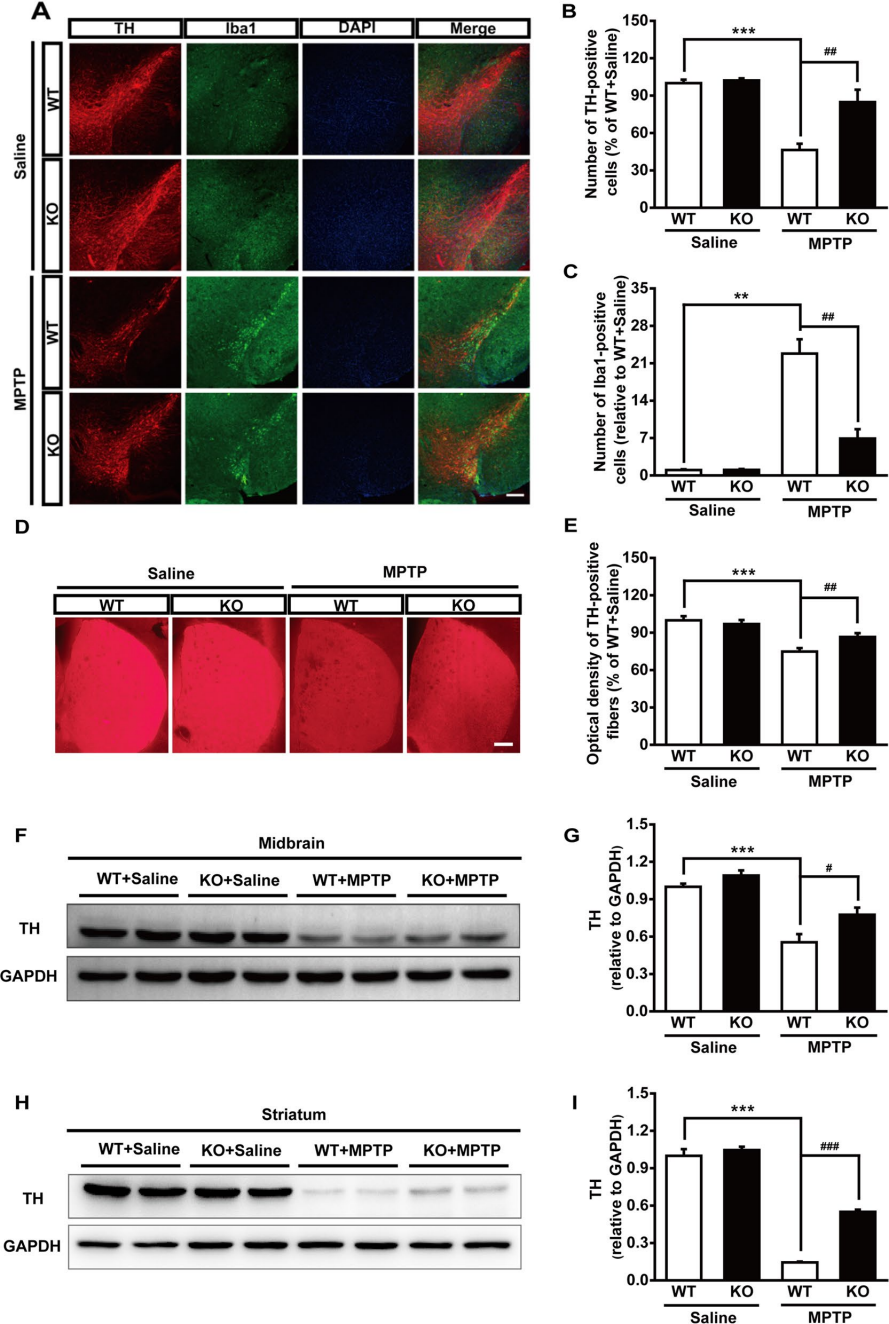
Genetic ablation of Maged1 rescues DA neurons from MPTP toxicity.** A** Immunofluorescence staining for TH (red) and Iba1 (green) in the substantia nigra of WT or Maged1 KO mice (induced or not induced with MPTP), nuclei were counterstained with DAPI (blue).Scale bar: 200 μm. **B**, **C** Quantification of TH-positive cells (**B**) or Iba1-positive cells (**C**) in the substantia nigra (% of WT + saline). **D** Representative images of TH-positive fibers in striatum sections. Scale bar: 200 μm**. E** Quantitative analysis of the optical density of TH-positive fibers in (**D**) using ImageJ software. **F**, **G** Western blot analysis illustrating the expression of TH in the midbrain; GAPDH was used as a loading control. **H**, **I** Western blot analysis illustrating the expression of TH in the striatum; GAPDH was used as a loading control. For **A**–**E**, WT + saline: n = 4, KO + saline: n = 4, WT + MPTP: n = 6, KO + MPTP: n = 5. For **F**–**I**, n = 4 for each group. Data are shown as means ± SE. **P < 0.01, ***P < 0.001, ^#^*P* < 0.05, ^##^*P* < 0.01, ^###^*P* < 0.001

It has been well known that MPTP treatment of mice contributed to the damage to the nigrostriatal pathway resulting in a specific loss of nigral DA neurons and a profound reduction of dopaminergic nerve fibers projecting to the striatum. In this study, we also observed that MPTP treatment led to an approximately 54% loss of TH-positive staining in the SN (Fig. 3A, B) and a similar loss of dopaminergic innervation was observed in the striatum (Fig. 3D, E). While, Maged1 deficit protected mice from the MPTP-induced DA neurodegeneration in the SN (Fig. 3A, B) and in the striatum (Fig. 3D, E). Similar results were confirmed using western blot analysis for TH protein levels in the midbrain (Fig. 3F, G) and striatum (Fig. 3H, I). Moreover, MPTP-treated mice usually develop rapid microgliosis in the striatum and SN [22]. Interestingly, we found that the expression of Iba1 (a marker of microglia) in the SN was significantly attenuated in MPTP-treated Maged1 KO mice (Fig. 3A and C). In addition, immunolabeling using the α-Synuclein antibody showed the significant α-Synuclein accumulation in the SN of MPTP-treated WT mice (Additional file 1: Fig. S1). In contrast, only mild α-Synuclein immunoreactivity was detected in the SN of MPTP-treated Maged1 KO mice (Additional file 1: Fig. S1). These results suggested that Maged1 knockout could attenuate MPTP-induced neurotoxicity in the nigrostriatal pathway in mice.

### Protective effect of Maged1 deficiency against MPP^+^ on primary DA neurons and differentiated ReNcell VM cells

**Fig. 4:**
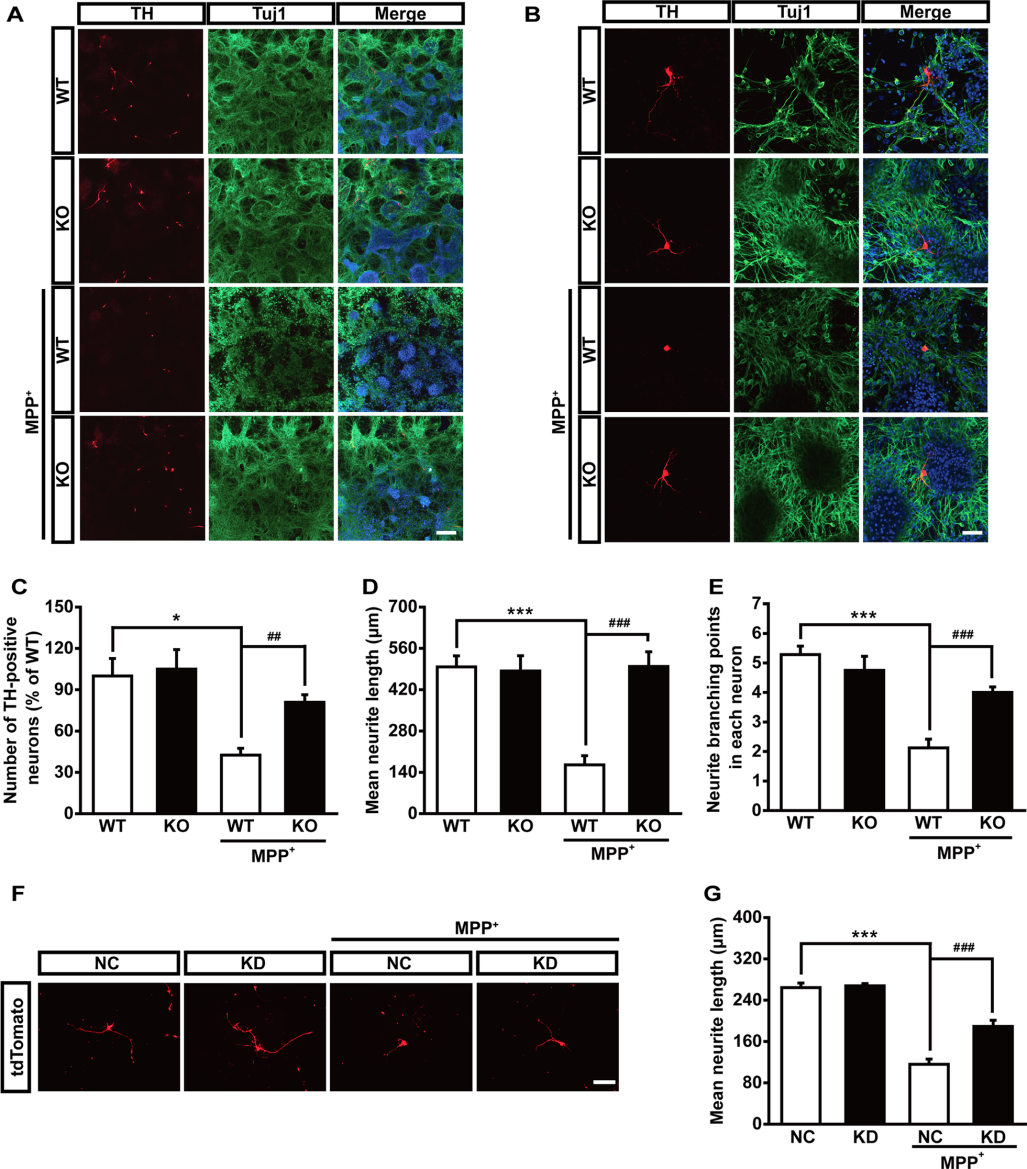
Maged1 deficiency protects dopaminergic neurons against MPP^+^-induced toxicity.** A** Double-label immunocytochemistry for TH (red) and Tuj1 (green) in primary mesencephalic culture; nuclei were counterstained with DAPI (blue). Scale bar: 200 μm. **B** High-magnification images of (**A**). Scale bar: 50 μm. **C**–**E** The number of TH-positive dopaminergic neurons (**C**, n = 6 different fields), mean neurite length (μm) (**D**, n = 12 TH-positive cells), and neurite branching points in each neuron (**E**, n = 12 TH-positive cells) in (**A**, **B**). **F** ReNcell VM cells were stably transduced with packaged lentivirus vectors to express tdTomato and differentiated for 12 days followed by NC or si-Maged1 transfection; representative images were acquired after treatment with 200 μM MPP^+^ for 24 h. Scale bar: 100 μm. **G** Measurements of neurite length from (**G**, n = 12 tdTomato-labeled cells). Data are shown as means ± SE. * *P* < 0.05, *** *P* < 0.001, ^##^*P* < 0.01, ^###^*P* < 0.001. *NC* normal control, *KD* knock down

To further confirm the protective effect of Maged1 deficiency against MPP^+^ in vitro, we isolated and cultured the primary mesencephalic neurons from E13.5 mouse embryos of WT or Maged1 KO mice. The deficiency of Maged1 significantly reduced the MPP^+^-induced loss of TH-immunoreactive mouse primary mesencephalic neurons in culture (Fig. 4A and C). Moreover, Maged1 deficiency counteracted MPP^+^-induced shortening of neurite length and the reduced branching in the primary dopaminergic cultures of the mouse midbrain tissue (Fig. 4B, D and E). Moreover, ReNcell VM cells, a human neural progenitor cell line, were applied to further investigate the role of Maged1 in DA neurons. We first differentiated ReNcell VM cells into dopaminergic neurons and further transfected the cells with Maged1-siRNA to inhibit endogenous Maged1. Consistent with the above data, MPP^+^ exposure resulted in significantly shorter neurites in differentiated ReNcell VM cells, which was partly rescued in cells transfected with siRNA-Maged1 (Fig. 4F, G). Collectively, our data demonstrated that lack of Maged1 attenuated the neurotoxic effect of MPP^+^ on cultured cells in vitro.

### Maged1 deficiency protects dopaminergic neurons against cell apoptosis triggered by MPTP or MPP^+^

**Fig. 5:**
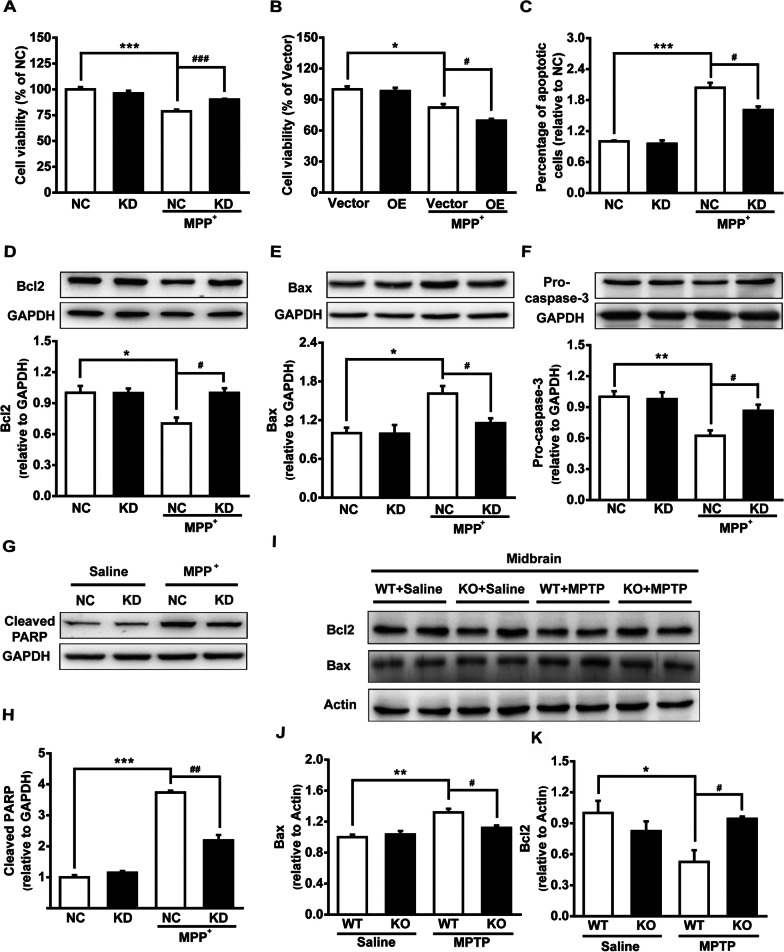
Maged1 deficiency suppresses cell apoptosis in MPP^+^-intoxicated cells and MPTP-treated mice.** A** SH-SY5Y cells were transfected with NC or si-Maged1; after 36 h, cells were treated with 200 μM MPP^+^ for 48 h and cell viability quantified using the CCK8 assay. **B** SH-SY5Y cells were transfected with Vector or Maged1-HA; after 24 h, cells were treated with 200 μM MPP^+^ for 48 h and cell viability was quantified using the CCK8 assay. **C** SH-SY5Y cells transfected with NC or si-Maged1 were treated with 200 μM MPP^+^ for 48 h, apoptotic cells were stained using PI, measured using flow cytometry, and quantified. **D** SH-SY5Y cells were transfected with NC or si-Maged1 and treated with 200 μM MPP^+^ for 48 h and cell lysates were detected for Bcl-2, Bax, Pro-caspase-3, and Cleaved PARP; GAPDH was used as a loading control. **E**–**H** Quantification of Bcl2/GAPDH (**E**), Bax/GAPDH (**F**), Pro-caspase-3/GAPDH (**G**), and Cleaved PARP/GAPDH (**H**) from results of (**D**). **I** Original bands of Bcl2 and Bax from midbrain extracts; actin was used as a loading control. **J**, **K** Quantification of Bcl2/Actin (**J**) and Bax/Actin (**K**) from the results of (**I**), n = 4 for each group. For **A**–**H**, results are representative of three separate experiments. Data are shown as means ± SE. *P < 0.05, **P < 0.01, ***P < 0.001, ^#^*P* < 0.05, ^##^*P* < 0.01, ^###^*P* < 0.001. *NC* normal control, *KD* knock down

MPTP-mediated apoptosis of DA neurons has been considered to be a major cause of neuronal death. Given that Maged1 plays an important role in neuronal apoptosis, we hypothesized that Maged1 was involved in MPP^+^-induced apoptosis. Thus, SH-SY5Y cells were transfected with Maged1-siRNA or Maged1-HA to selectively modulate the expression of Maged1 and were further treated using MPP^+^. Cell viability was assessed using a CCK8 assay. As shown in Fig. 5, MPP^+^ treatment reduced the cell viability in control SH-SY5Y cells, which was attenuated by Maged1-siRNA transfection, while that was aggravated by overexpression with Maged1-HA (Fig. 5A, B). Furthermore, apoptotic cells were quantified using flow cytometry. Exposure to MPP^+^ induced noticeable apoptosis in SH-SY5Y cells, which was effectively blocked by Maged1-siRNA transfection (Fig. 5C). We then investigated this protective effect of Maged1 knockdown by examining the apoptosis-related proteins. As shown in Fig. 5D–H, MPP^+^ treatment resulted in the increased expression of Bax and the cleaved PARP, as well as decreased the expression of Bcl2 and pro-caspase-3, and all of these effects were blocked by Maged1-siRNA transfection in cultured SH-SY5Y cells, indicating that apoptosis was greatly reversed by Maged1 knockdown. Similarly, we observed the increased Bax and decreased Bcl2 expression in the midbrain of MPTP-treated mice, and which was rescued in Maged1 knockout mice (Fig. 5I–K). Taken together, these results suggested that elevated Maged1 may play a critical role in MPTP- or MPP^+^-induced cell apoptosis while Maged1 knockdown could block this neurotoxic insult.

### Maged1 deficiency promoted autophagy in dopaminergic neurons

**Fig. 6:**
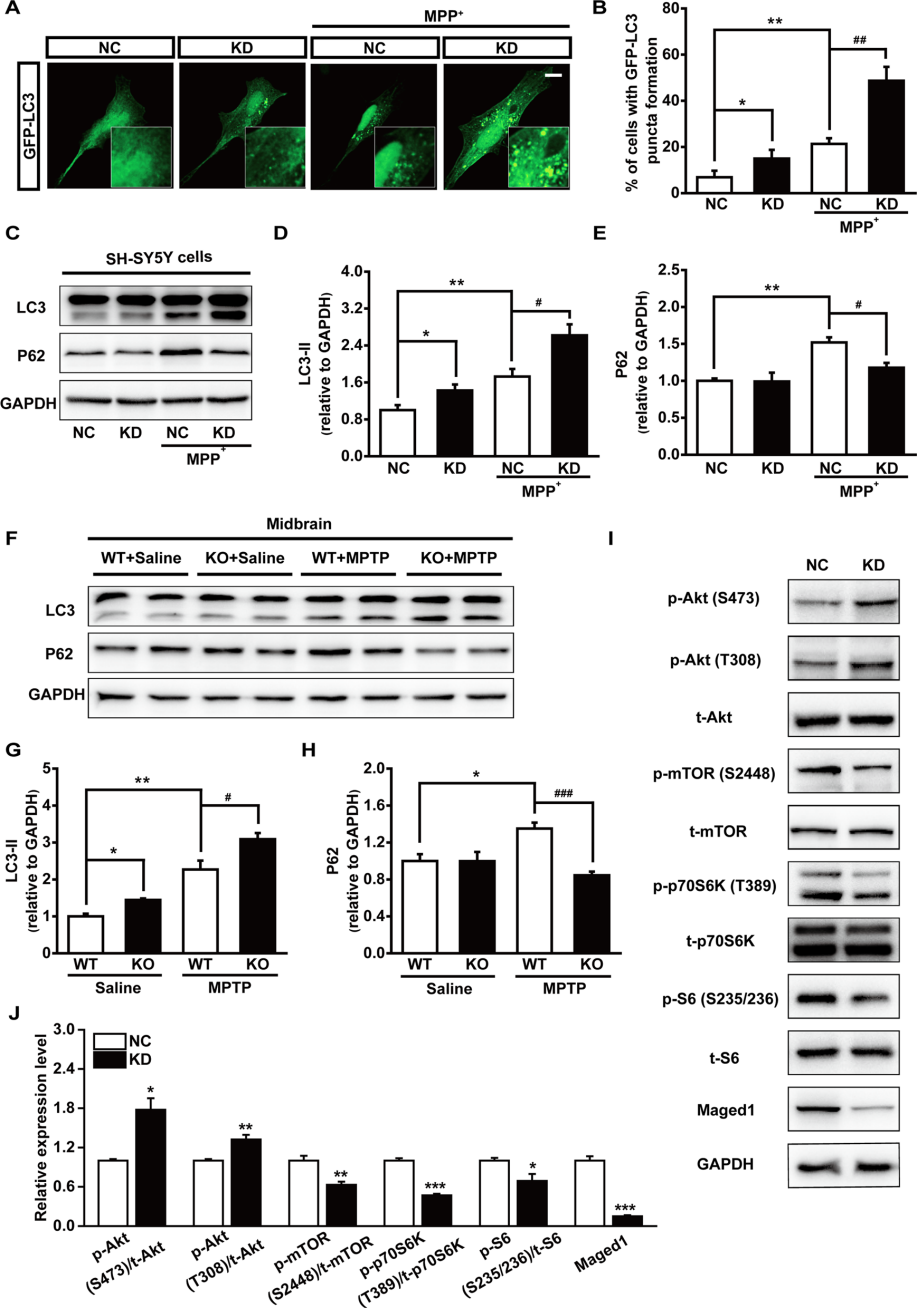
Maged1 deficiency attenuates impairment of autophagy in MPP^+^-intoxicated cells and MPTP-treated mice**. A** SH-SY5Y cells were transfected with GFP-LC3 and visualized using confocal microscopy. Scale bar:10 μm. **B** Quantification of GFP-LC3 puncta formation in (**A**), n = 6 different fields. **C** SH-SY5Y cells were transfected with NC or si-Maged1 and treated with 200 μM MPP^+^ for 48 h and cell lysates were detected for LC3 and P62. **D**, **E** Quantification of LC3II/GAPDH (**D**) and P62/GAPDH (**E**) from the results of (**C**). **F** Three days after the final administration of MPTP, mice midbrains were collected and protein expression of LC3 and P62 was measured using western blot; GAPDH was used as a loading control. **G**, **H** Quantification of LC3II/GAPDH (G) and P62/GAPDH (**H**) from results of (**F**), n = 4 for each group. **I**, **J** Western blot analysis was used to detect protein levels of p-Akt (S473), p-Akt (T308), t-Akt, p-mTOR (S2448), t-mTOR, p-p70S6K (T389), t-p70S6K, p-S6 (S235/236), t-S6, and Maged1 in NC or si-Maged1-transfected SH-SY5Y cells. For A-I, experiments were performed in triplicate. Data are shown as means ± SE. * *P* < 0.05, ** *P* < 0.01, *** *P* < 0.001, ^#^*P* < 0.05, ^##^*P* < 0.01, ^###^*P* < 0.001. *NC* normal control, *KD* knock down

MPTP-induced autophagy is involved in regulating neuronal cell death. The neuroprotective effect of autophagy activation was determined in MPTP-treated mice and MPP^+^-intoxicated SH-SY5Y cells [23]. Next, we investigated whether Maged1 deficiency had pro-autophagic effects in dopaminergic neurons. Thus, we first transfected a GFP-LC3 vector in SH-SY5Y cells to visualize autophagosomes. As shown in Fig. 6A, B both the MPP^+^ treatment and Maged1 knockdown increased the autophagosome formation in SH-SY5Y cells. On the other hand, the Maged1-siRNA transfection increased the autophagosome formation compared with the control-siRNA transfection following MPP^+^ exposure, suggesting that Maged1 knockdown enhanced MPP^+^-induced autophagy. Then, we examined LC3 conversion and p62 levels, which are key components of autophagy, using western blot. Our results showed that LC3-II expression was upregulated and p62 expression was downregulated by Maged1 knockdown following MPP^+^ treatment (Fig. 6C–E). Identifying the autophagosome using transmission electron microscopy also showed that Maged1 knockdown increased autophagosomes in SH-SY5Y cells (Additional file 1: Fig. S2). To detect the alteration of autophagy in vivo, we collected the midbrain or striatum at different time after MPTP treatment, and analyzed the expression of LC3 and P62 using western blot. Results showed that relatively higher expression of both LC3 and P62 was detected after MPTP treatment for 3 days either in the midbrain or striatum, while it was more notable in the midbrain, indicating that the impaired autophagic flux in the midbrain could be detected after MPTP treatment for 3 days (Additional file 1: Fig. S3). Accordingly, results of the western blot (Fig. 6F–H) and p62 immunofluorescent staining (Additional file 1: Fig. S4) showed that the autophagic flux in the midbrain of 3-days-MPTP-treated mice was impaired in the wildtype group while was restored in Maged1 knockout group. These data indicated that Maged1 deficiency could enhance autophagic flux and autophagosome degradation impaired by MPTP or MPP^+^.

Previous studies have demonstrated that Maged1 facilitates cell survival by activating Akt signaling pathways in PC12 cells. Here, western blot analysis for the elevated Akt phosphorylation at both Thr308 and Ser473 substantiated the observation that Maged1 knockdown activated Akt in SH-SY5Y cells (Fig. 6I, J), implying that this regulatory pathway is also responsible for the anti-apoptotic effect against MPTP. To unravel the possible mechanisms underlying the autophagy modulation, we examined the target of rapamycin (TOR) kinase pathway, which is the main regulator of autophagy. We found that Maged1 knockdown significantly decreased the phosphorylation levels of mTOR (S2448), p70S6K (T389), and S6 (S235/236) proteins in SH-SY5Y cells (Fig. 6I, J), suggesting that mTORC1/S6K signaling pathway inhibition might be responsible for Maged1 deficiency-induced autophagy activation. Taken together, these results suggested that Maged1 knockdown in DA neurons might promote cell survival through the activation of Akt and activate autophagy through the inhibition of the mTOR kinase pathway.

### Aging-associated degeneration of DA neurons is attenuated in Maged1 knockout mice

**Fig. 7:**
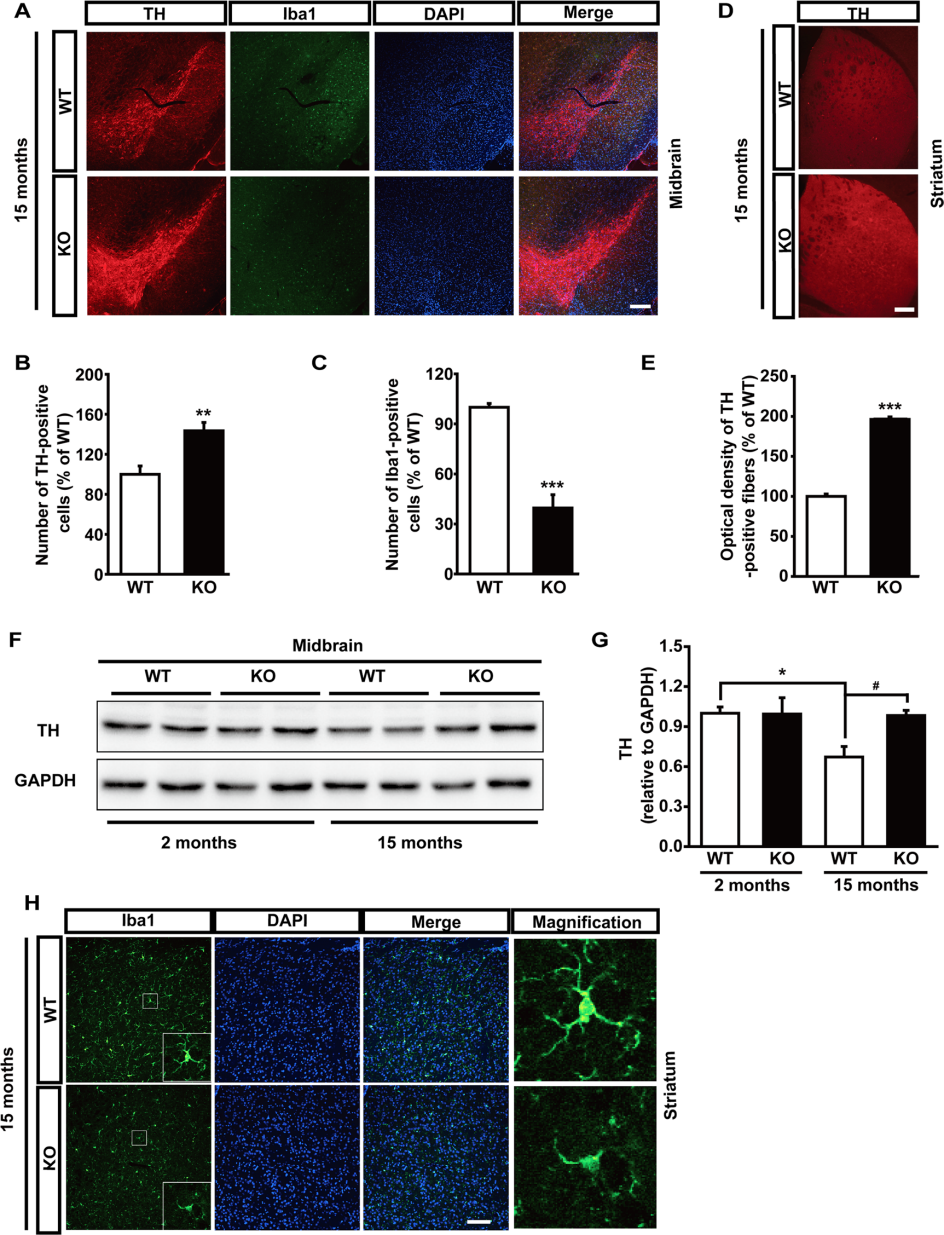
Maged1 knockout in mice prevents dopaminergic neurodegeneration in elderly mice.** A** Typical immunostaining for TH (red), Iba1 (green), and DAPI (blue) in the midbrain of 15-month-old mice. Scale bar: 200 μm. **B**, **C** Quantification of TH-positive cells **B** or Iba1-positive cells **C** in A (% of WT). **D** Representative images of TH-positive fibers in striatum sections. Scale bar: 200 μm. **E** Quantitative of the optical density of TH-positive fibers in **D** using ImageJ software. **F**, **G** Western blot analysis of TH expression from the midbrain of 2-month-old and 15-month-old mice. **H** Iba1 immunostaining in the striatum of 15-month-old mice. Scale bar: 200 μm. For **A**–**H**, n = 4 mice per group. Data are shown as means ± SE. *P < 0.05, **P < 0.01, ***P < 0.001, ^#^*P* < 0.05

Aging affects many cellular processes that trigger neurodegeneration and contributes to the pathogenesis of PD. We investigated whether Maged1 knockout could diminish PD-like pathology in aging mice. Immunofluorescence of TH revealed that Maged1 knockout preserved the nigral TH-positive neurons (Fig. 7A, B and striatal TH-positive fibers (Fig. 7D, E) in 15-month Maged1 KO mice. Noticeably, western blot showed that TH levels were reduced in the midbrain of 15-month-old mice compared to that in 2-month-old mice; however, such age-associated changes were not evident in the KO mice (Fig. 7F, G), suggesting that aging is the most prominent risk factor for PD and that Maged1 knockout could block age-related DA degeneration. Neuroinflammation is a hallmark of PD. We noticed that Maged1 knockout decreased microglial-specific Iba1 staining intensity in both the SN (Fig. 7A and C) and striatum (Fig. 7H), suggesting the alleviated neuroinflammation in the elderly Maged1 knockout mice. Overall, these findings indicated that Maged1 knockout attenuated dopaminergic degeneration pathology in the elderly mice.

## Discussion

PD is characterized by the progressive and selective degeneration of nigrostriatal DA neurons. There is currently no established neuroprotective or neurorestorative treatment for PD. In this study, we report that Maged1 is implicated in MPTP-induced neuronal toxicity. We observed that Maged1 knockout mice were more resistant to neurotoxin-induced DA neurodegeneration and motor deficits in a murine model of PD. We then confirmed that Maged1-deficient cells showed a similar protective phenotype against MPP^+^-induced toxicity in vitro. The ameliorating effects seen in Maged1-deficient mice or cells were highly correlated with the inhibition of apoptosis and enhancement of autophagy. Our study revealed that the upregulation of the Akt signaling pathway and downregulation of the mTOR signaling pathway might have been partly responsible for this protective effect of Maged1 ablation. Based on these observations, we suggest that elevated expression of Maged1 induced by MPTP disturbed the balance between apoptosis and autophagy and ultimately led to DA neurodegeneration.

The role of Maged1 in apoptosis has been well-studied. The neurotrophin receptor, p75NTR, plays an important physiological role in regulating cell death of neurons during neuronal development and is also implicated in the pathology of neurodegenerative diseases [18, 24–27]. Maged1 interacts with p75NTR, resulting in caspase activation and cell death through a JNK-dependent mitochondrial pathway [28]. In addition, Maged1 is reported to up-regulate p53 transcriptional activity [29], which is a well-known regulator of cell apoptosis and ferroptosis involved in PD [30, 31]. Thus, Maged1 may play a pro-apoptotic role through interacting with p75NTR or regulating the p53-signaling pathway in the progression of PD. Moreover, we demonstrated that Maged1 knockdown in SH-SY5Y cells resulted in the activation of the pro-survival kinase, Akt. It is reported that Akt activity is reduced in the striatum of patients with PD, suggesting that its inactivation has an important role in PD [32]; being so, the up-regulation of the Akt-signaling pathway induced by Maged1 deficiency may also contribute to the protective effects of Maged1 knockout in PD models.

Dysfunctional autophagy plays a vital role in the pathogenesis of PD [33]. Aggregation of autophagosomes or lysosomal depletion has been observed both in the SN from the postmortem brains of PD patients and PD animal models [34]. However, there is still no report about whether Maged1 can regulate autophagy. In this study, we reported that Maged1 deficiency can induce autophagy in PD models both in vivo and in vitro. Our study demonstrated that Maged1 knockdown increased the number of autophagosomes and expression of LC3-II in SH-SY5Y cells with or without MPP^+^ treatment. The mTOR signaling pathway is one of the most important mechanisms of autophagy in cells; mTOR plays a complex role in the induction, progress, and termination of autophagy [11]. To further elucidate the molecular mechanism of Maged1 deficiency-induced autophagy in SH-SY5Y cells, some key proteins in the mTOR signaling pathway were studied. Results showed that Maged1 knockdown inhibited the phosphorylation of mTOR and its downstream target protein, S6 kinase and p70 ribosomal S6 kinase (p70/S6K), to increase autophagy. In addition, apoptosis can be suppressed by Akt activation through the negative feedback loop from p70/S6K [35], and the activation of the mTOR/p70/S6K axis can promote protein synthesis and cell survival [36]. Therefore, Maged1 could play an important role in the balance of cell apoptosis and autophagy in PD.

Studies report that Maged1 has multiple functions in brain development and function. Reddy et al. have shown that Maged1 interacts with the TrkA receptor and plays an important role in the differentiation and survival of neuronal progenitor cells [37]. Sasaki et al. have reported that Maged1 could regulate Dlx/Msx-dependent transcriptional activity and control the development differentiation and migration of GABAergic neurons [38]. Williams et al. showed that Maged1 may act as an adaptor protein for receptors, such as UNC5H and p75NTR, to mediate programmed neuronal cell death [20]. More importantly, Maged1 regulates a large variety of behaviors and has been implicated in psychiatric disorders, including regulation of circadian rhythms [39], social behavior [40], depression [41], memory formation [17] and cocaine addiction [42]. Here, we report that Maged1 deficiency showed a neuroprotective effect on a neurotoxin-induced PD model through apoptosis inhibition and autophagy enhancement. Our results showed that Maged1 played a key role in the pathogenesis of PD and that inhibition of Maged1 might be a promising therapeutic drug target for PD. However, the complicated function of Maged1 in the central nervous system could pose a challenge to develop specific therapeutic drugs in the management of PD by targeting Maged1.

We acknowledge several limitations to this work. Firstly, we used conventional Maged1 knockout mice in our in vivo studies. Future studies could, therefore, use a DA neuron-specific knockout mouse model to exclude the influence of other cell types. Secondly, acute MPTP administration to mice was used in our study to mimic the PD model. Alternatively, a chronic MPTP model of PD or a 6-OHDA PD model would shed more light for future studies. Lastly, we presented here that both activated Akt signaling and inhibited mTORC1/S6K signaling pathway might be responsible for the beneficial effects of Maged1 deficiency in the PD model. It seemed mechanistically paradoxical, but indicated that Maged1 might play a pivotal role in the balance of cell apoptosis (Akt signaling dependent) and autophagy (mTORC1/S6K signaling dependent) in PD. Previous studies have revealed that Maged1 mediates multiple functions by interacting with different proteins [16], here it would be clinically and pharmaceutically insightful to determine its precise mechanism in the future studies.

### Supplementary Information

**Additional file 1: Figure S1.** A Immunofluorescence staining for α-Synuclein in the substantia nigra derived from WT or Maged1 KO mice with or without MPTP treatment. Scale bar: 200 μm. B Quantification of α-Synuclein fluorescence intensity in the substantia nigra (% of WT+saline), WT+saline: n=4, KO+saline: n=4, WT+MPTP: n=6, KO+MPTP: n=5. *P < 0.05, ^#^P < 0.05. **Figure S2.** SH-SY5Y cells were transfected with NC or si-Maged1 and autophagosome was detected using transmission electron microscopy (TEM). Scale bar: 1 μm. Black arrow 2 indicates autophagosome. NC: normal control, KD: knock down. **Figure S3.** A, B Western blot analyses illustrating the expression of LC3 and P62 in midbrain A and striatum B at different time points after MPTP treatment. **Figure S4.** Immunofluorescence staining for TH (red) and P62 (green) in the substantia nigra derived from WT or Maged1 KO mice (induced or not induced with MPTP), nuclei were counterstained with DAPI (blue). Scale bar: 50 μm. White arrow indicates P62-aggregated TH-positive neuron. **Table S1.** Antibodies. **Table S2.** Reagents.

## Data Availability

The data that support the findings of this study are available from the corresponding author upon reasonable request.
