# Correction: The deficiency of Maged1 attenuates Parkinson’s disease progression in mice

**DOI:** 10.1186/s13041-023-01075-1

**Published:** 2024-02-09

**Authors:** Jie Wang, Sheng‑Ye Xu, Zhi‑Yuan Ye, Zhou‑Na Sun, Jia‑Qi Zhang, Cui Qi, Rui Liu, Xiang Gao, Chuan He, Wei‑Yan You, Jun Gao

**Affiliations:** 1 Department of Neurobiology, School of Basic Medical Sciences, Nanjing Medical University, Nanjing, China; 2 Department of Rehabilitation Medicine, The Affiliated Jiangsu Shengze Hospital of Nanjing Medical University, Nanjing Medical University, Nanjing, China; 3 SKL of Pharmaceutical Biotechnology and Model Animal Research Center, Collaborative Innovation Center for Genetics and Development, Nanjing Biomedical Research Institute, Nanjing University, Nanjing, 210061 China

**Correction: Molecular Brain (2023) 16:22** 10.1186/s13041-023-01011-3

Following publication of the original article [1], the authors identified an error in Fig. 3a. The images of KO mice for Saline group in Fig. 3A were mistakenly assembled. The authors have carefully checked the raw data and identified that this was a case of clerical error. The correct figure and caption is given hereafter.

The incorrect Fig. 3:

**Fig. 3:**
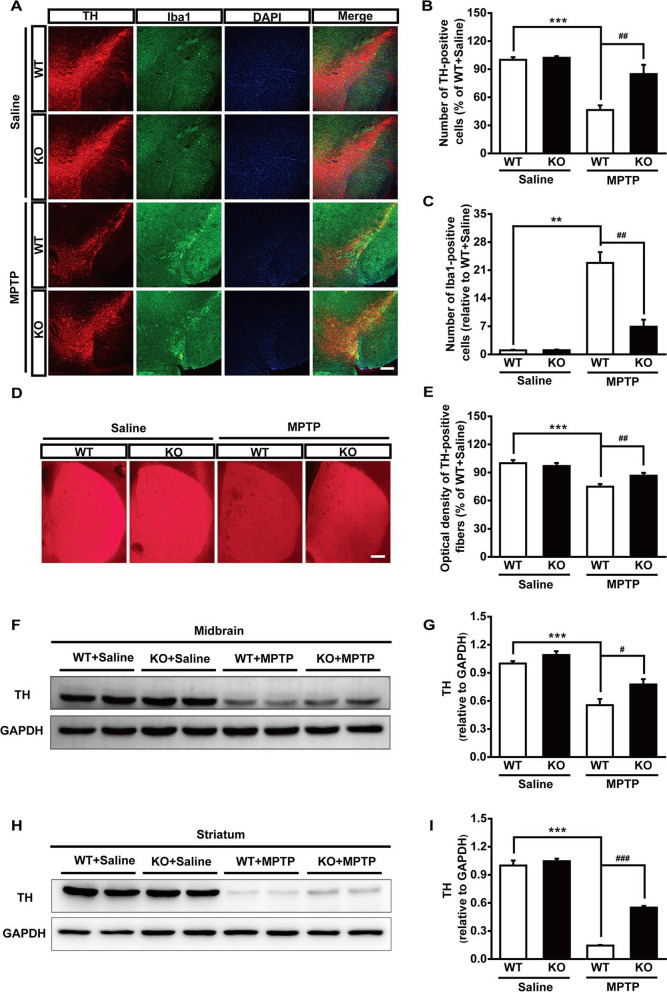
Genetic ablation of Maged1 rescues DA neurons from MPTP toxicity. **A** Immunofluorescence staining for TH (red) and Iba1 (green) in the substantia nigra of WT or Maged1 KO mice (induced or not induced with MPTP), nuclei were counterstained with DAPI (blue).Scale bar: 200 μm. **B**, **C** Quantification of TH-positive cells (**B**) or Iba1-positive cells (**C**) in the substantia nigra (% of WT + saline). **D** Representative images of TH-positive fibers in striatum sections. Scale bar: 200 μm. **E** Quantitative analysis of the optical density of TH-positive fibers in (**D**) using ImageJ software. **F**, **G** Western blot analysis illustrating the expression of TH in the midbrain; GAPDH was used as a loading control. **H**, **I** Western blot analysis illustrating the expression of TH in the striatum; GAPDH was used as a loading control. For **A–E**, WT + saline: n = 4, KO + saline: n = 4, WT + MPTP: n = 6, KO + MPTP: n = 5. For **F–I**, n = 4 for each group. Data are shown as means ± SE. **P < 0.01, ***P < 0.001, ^#^*P* < 0.05, ^##^*P* < 0.01, ^###^*P* < 0.001.

The correct Fig. 3:

**Fig. 3:**
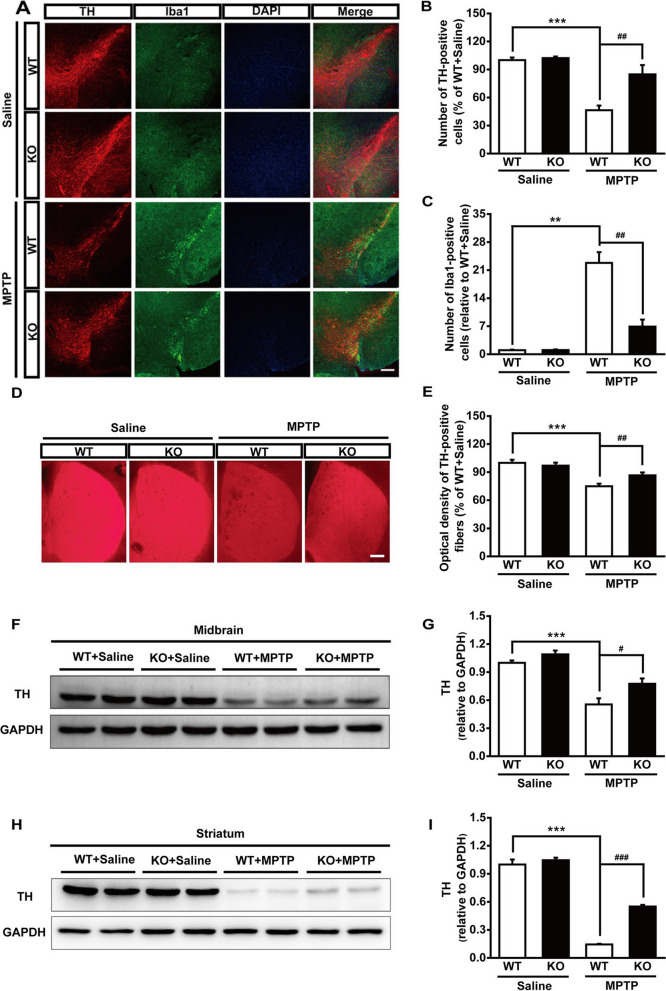
Genetic ablation of Maged1 rescues DA neurons from MPTP toxicity. **A** Immunofluorescence staining for TH (red) and Iba1 (green) in the substantia nigra of WT or Maged1 KO mice (induced or not induced with MPTP), nuclei were counterstained with DAPI (blue).Scale bar: 200 μm. **B**, **C** Quantification of TH-positive cells (**B**) or Iba1-positive cells (**C**) in the substantia nigra (% of WT + saline). **D** Representative images of TH-positive fibers in striatum sections. Scale bar: 200 μm. **E** Quantitative analysis of the optical density of TH-positive fibers in (**D**) using ImageJ software. **F**, **G** Western blot analysis illustrating the expression of TH in the midbrain; GAPDH was used as a loading control. **H**, **I** Western blot analysis illustrating the expression of TH in the striatum; GAPDH was used as a loading control. For **A–E**, WT + saline: n = 4, KO + saline: n = 4, WT + MPTP: n = 6, KO + MPTP: n = 5. For **F–I**, n = 4 for each group. Data are shown as means ± SE. **P < 0.01, ***P < 0.001, ^#^*P* < 0.05, ^##^*P* < 0.01, ^###^*P* < 0.001.

Figure 3a has been updated above and the original article [1] has been corrected.
