# Supplementary material for: The deficiency of Maged1 attenuates Parkinson's disease progression in mice
Source: Mol Brain. 2023 Feb 11;16:22. doi: 10.1186/s13041-023-01011-3 (PMC9921624; doi:10.1186/s13041-023-01011-3)
Supplement: Supplementary file 1 — Additional file 1: Figure S1. A Immunofluorescence staining for α-Synuclein in the substantia nigra derived from WT or Maged1 KO mice with or without MPTP treatment. Scale bar: 200 μm. B Quantification of α-Synuclein fluorescence intensity in the substantia nigra (% of WT+saline), WT+saline: n=4, KO+saline: n=4, WT+MPTP: n=6, KO+MPTP: n=5. *P < 0.05, #P < 0.05. Figure S2. SH-SY5Y cells were transfected with NC or si-Maged1 and autophagosome was detected using transmission electron microscopy (TEM). Scale bar: 1 μm. Black arrow 2 indicates autophagosome. NC: normal control, KD: knock down. Figure S3. A, B Western blot analyses illustrating the expression of LC3 and P62 in midbrain A and striatum B at different time points after MPTP treatment. Figure S4. Immunofluorescence staining for TH (red) and P62 (green) in the substantia nigra derived from WT or Maged1 KO mice (induced or not induced with MPTP), nuclei were counterstained with DAPI (blue). Scale bar: 50 μm. White arrow indicates P62-aggregated TH-positive neuron. Table S1. Antibodies. Table S2. Reagents. [file 13041_2023_1011_MOESM1_ESM.pdf]

Online supplement

The deficiency of Maged1 attenuates Parkinson's disease progression by inhibiting apoptosis and enhancing autophagy in mice

Content:

Figures S1-S4

Tables S1-S2

Supplemental Figures

Figure S1

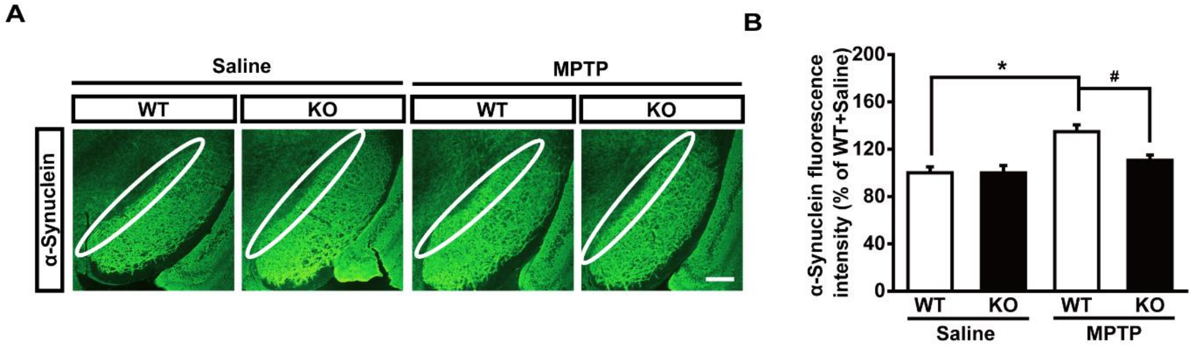

Figure S1. (A) Immunofluorescence staining for α-Synuclein in the substantia nigra derived from WT or Maged1 KO mice with or without MPTP treatment. Scale bar: 200 μm. **(B)** Quantification of α-Synuclein fluorescence intensity in the substantia nigra (% of WT+saline), WT+saline: n=4, KO+saline: n=4, WT+MPTP: n=6, KO+MPTP: n=5. * $P < 0.05$, $^{\#} P < 0.05$.

Figure S2

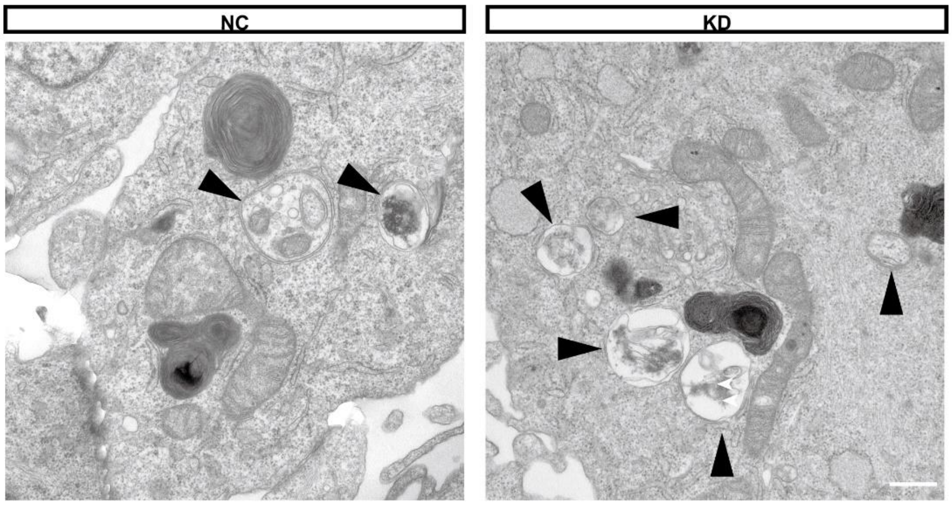

Figure S2. SH-SY5Y cells were transfected with NC or si-Maged1 and autophagosome was detected using transmission electron microscopy (TEM). Scale bar: 1 μm. Black arrow

indicates autophagosome. NC: normal control, KD: knock down.

Figure S3

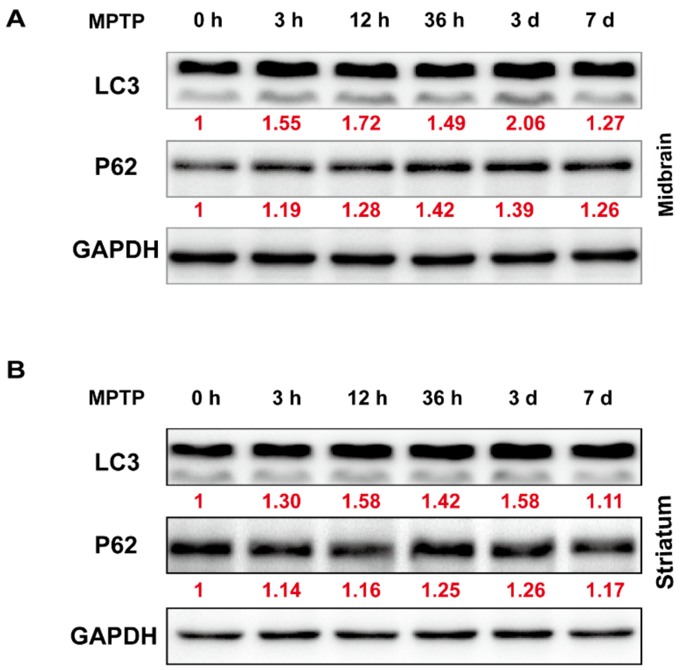

Figure S3. (A and B) Western blot analyses illustrating the expression of LC3 and P62 in midbrain (A) and striatum (B) at different time points after MPTP treatment.

Figure S4

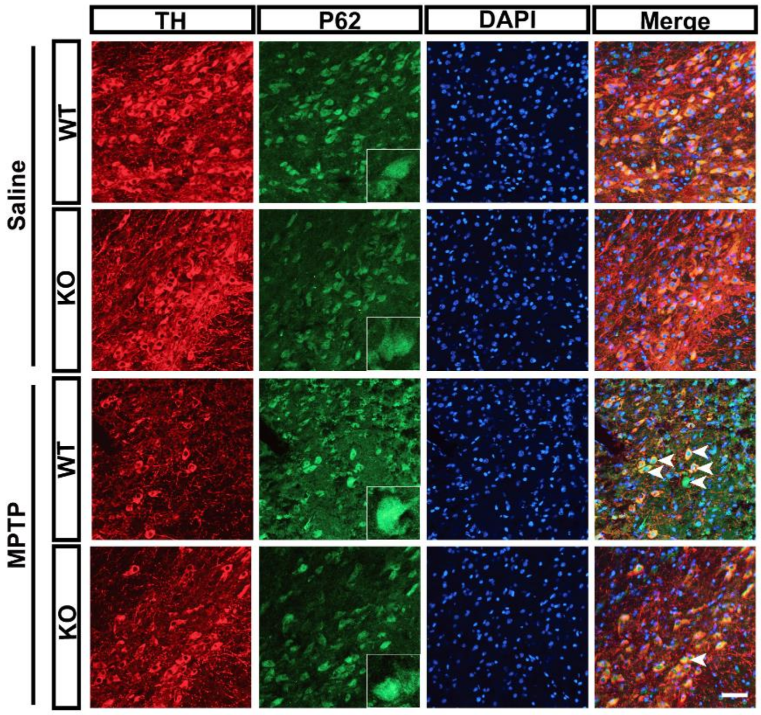

Figure S4. Immunofluorescence staining for TH (red) and P62 (green) in the substantia nigra derived from WT or Maged1 KO mice (induced or not induced with MPTP), nuclei were counterstained with DAPI (blue). Scale bar: 50 μm. White arrow indicates P62-aggregated TH-positive neuron.

Supplemental Tables

Table S1. Antibodies

Target antigen	Vendor or Source	Catalog	Working
Maged1	Proteintech	22053-1-AP	1:2000 for WB;
P62/SQSTM1	Proteintech	18420-1-AP	1:1000 for WB;
Bcl2	Proteintech	12789-1-AP	1:1000 for WB
LC3A/B	Cell Signaling Technology	12741#	1:1000 for WB
α-synuclein	Cell Signaling Technology	4179#	1:200 for IF
Cleaved PARP	Cell Signaling Technology	5625#	1:1000 for WB
Caspase-3	Cell Signaling Technology	9662#	1:1000 for WB
β3-Tubulin	Cell Signaling Technology	5568#	1:1000 for IF
Akt	Cell Signaling Technology	9272S#	1:1000 for WB
p-Akt (T308)	Cell Signaling Technology	9275S#	1:1000 for WB
p-Akt (S473)	Cell Signaling Technology	9271T#	1:1000 for WB
S6	Cell Signaling Technology	2317S#	1:1000 for WB
p-S6 (S235/236)	Cell Signaling Technology	2211S#	1:1000 for WB
p70S6K	Cell Signaling Technology	2708#	1:1000 for WB
p-p70S6K	Cell Signaling Technology	95796#	1:1000 for WB
mTOR	Cell Signaling Technology	2972#	1:1000 for WB
p-mTOR (S2448)	Cell Signaling Technology	2971#	1:1000 for WB
Tyrosine Hydroxylase	Sigma	T2928	1:1000 for WB;
Iba1	Wako	019-19741	1:1000 for IF
GFP	abcam	ab13970	1:200 for IF
Bax	Bioworld Technology	BS2538	1:1000 for WB
Actin	Bioworld Technology	BS6007MH	1:1000 for WB
GAPDH	Bioworld Technology	AP0063	1:1000 for WB

Goat Anti-Mouse IgG	Bioworld Technology	BS10006	1:400
Goat anti-Mouse IgG	Bioworld Technology	BS50350	1:10000
Goat anti-Rabbit IgG	Bioworld Technology	BS13278	1:10000
Donkey anti-Rabbit IgG (H+L) Cross-Adsorbed	Thermo Fisher	SA5-10038	1:1000
Alexa Fluor 488 AffiniPure Donkey Anti-	Jackson	703-545-155	1:400

Table S2. Reagents

Description	Source / Repository
RIPA	Sigma-Aldrich
DAPI	Sigma-Aldrich
CCK-8	Dojindo
DMEM/F12	KeyGEN BioTECH
MPP^+	Sigma
MPTP	Sigma
Laminin	Sigma
pentobarbital	Sigma
BCA protein quantitative kit	ThermoFisher
Lipofectamine-iMAX	ThermoFisher
Lipofectamine 2000	ThermoFisher
Protease Inhibitor Cocktail	Thermo Fisher
phosphatase inhibitors	Thermo Fisher
Accutase	ThermoFisher
B27	ThermoFisher
Glutamax	ThermoFisher
Penicillin and Streptomycin	ThermoFisher
bFGF-2	Peprotech
EGF	Peprotech
cAMP	Peprotech
GDNF	Peprotech
BCA Protein Assay kit	Beyotime
polyvinylidene difluoride membrane	Millipore
Neurobasal medium	ThermoFisher
